# COVID-19 - Endothelial Axis and Coronary Artery Bypass Graft Patency: a Target for Therapeutic Intervention?

**DOI:** 10.21470/1678-9741-2020-0303

**Published:** 2020

**Authors:** Gokce Topal, Andrzej Loesch, Michael R. Dashwood

**Affiliations:** 1 Department of Pharmacology, Faculty of Pharmacy, Istanbul University, Istanbul, Turkey.; 2 Centre for Rheumatology, Royal Free Hospital Campus, University College Medical School, London, United Kingdom.; 3 Department of Surgical and Interventional Sciences, Royal Free Hospital Campus, University College Medical School, London, United Kingdom.

**Keywords:** COVID-19, SARS Virus, Coronary Artery Bypass Graft Surgery, Saphenous Vein, Endothelial Dysfunction, Vascular Inflammation

## Abstract

It has been reported that severe acute respiratory syndrome coronavirus 2 (SARS-CoV-2) infection induces endothelial inflammation, therefore facilitating the progression of endothelial and vascular dysfunction in coronavirus disease 2019 (COVID-19) patients. Coronary artery bypass grafting (CABG) involves mainly the use of the saphenous vein (SV) and internal mammary artery as graft material in the stenosed coronary arteries. Unfortunately, graft patency of the SV is low due to endothelial dysfunction and inflammation. We propose that SARS-CoV-2 might cause vascular inflammation, endothelial dysfunction, and thrombosis in coronary artery bypass graft vessels by binding angiotensin-converting enzyme 2 receptor. Therefore, in this Special Article, we consider the potential influence of COVID-19 on the patency rates of coronary artery bypass graft vessels, mainly with reference to the SV. Moreover, we discuss the technique of SV graft harvesting and the therapeutic potential of focusing on endothelial dysfunction, vascular inflammation, and thrombosis for protecting coronary artery bypass grafts in COVID-19 infected CABG patients.

**Table:** 

Abbreviations, acronyms & symbols				
**A**	**= Artery**		**Lu**	**= Lumen**
**ACE**	**= Angiotensin-converting enzyme**		**NO**	**= Nitric oxide**
**ACE2**	**= Angiotensin-converting enzyme 2**		**NT**	**= No-touch**
**CABG**	**= Coronary artery bypass grafting**		**P**	**= Pericyte**
**CAD**	**= Coronary artery disease**		**PGI2**	**= Prostacyclin**
**Col**	**= Collagen**		**RAAS**	**= Renin-angiotensin-aldosterone system**
**COVID-19**	**= Coronavirus disease 2019**		**SARS-CoV-2**	**= Severe acute respiratory syndrome coronavirus 2**
**En**	**= Endothelium**		**Sm**	**= Vascular smooth muscle**
**ET-1**	**= Endothelin-1**		**SV**	**= Saphenous vein**
**Exm**	**= Extracellular matrix**		**TNFα**	**= Tumor necrosis factor alpha**
**IL-6**	**= Interleukin 6**		**V**	**= Vein**
**IMA**	**= Internal mammary artery**			

## INTRODUCTION

**Fig. 1:**
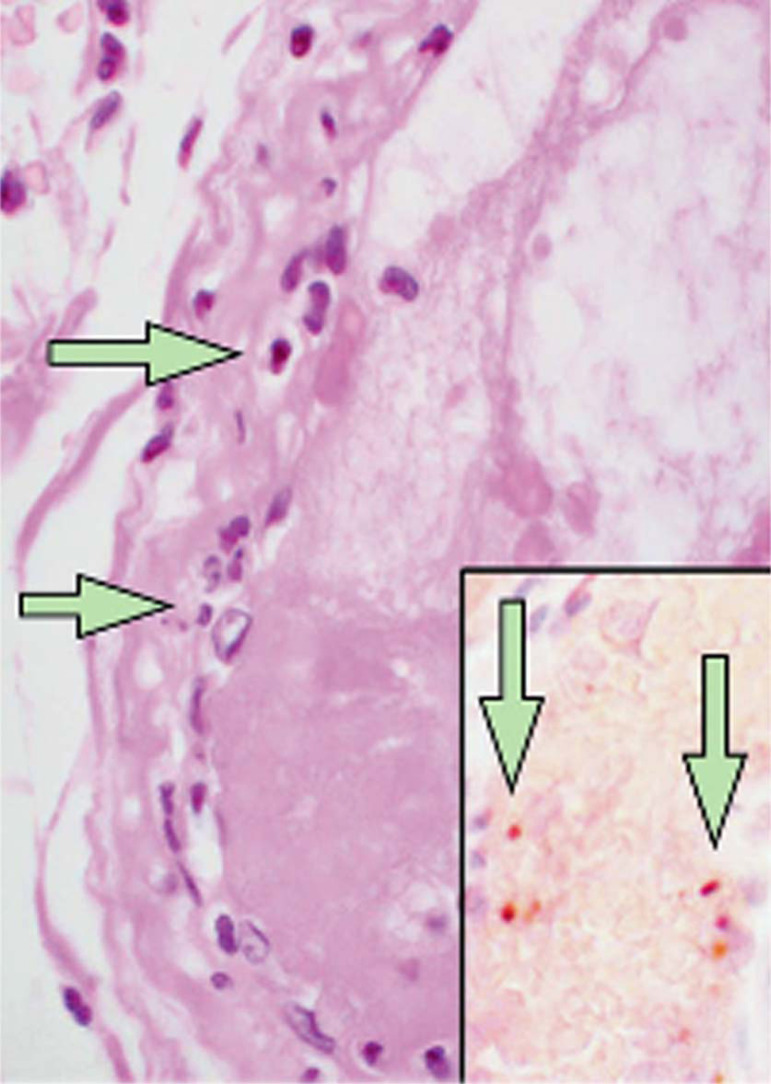
Small bowel resection specimen from a coronavirus disease 2019 (COVID-19) patient stained with haematoxylin and eosin. Arrows point to dominant mononuclear cell infiltrates within the intima along the lumen of many vessels. The inset shows an immunohistochemical staining of caspase 3 (enzyme implicated in cell apoptosis) in small bowel specimens indicating apoptosis of endothelial cells and mononuclear cells (From Varga et al.^[4]^, which is acknowledged).

There is increasing evidence demonstrating the impact of coronavirus disease 2019 (COVID-19) on cardiovascular function in patients with pre-existing cardiovascular disease^[1,2]^. Recently, Mehra et al.^[3]^ demonstrated and confirmed previous reports showing that an underlying cardiovascular disease (coronary artery disease [CAD], heart failure, and cardiac arrhythmias) is associated with an increased risk of in-hospital death among patients with COVID-19. Furthermore, evidence of direct viral infection of endothelial cells and diffuse endothelial inflammation by COVID-19 has been demonstrated, suggesting that severe acute respiratory syndrome coronavirus 2 (SARS-CoV-2) infection facilitates endothelial injury. An example of such pathological changes showing accumulation of inflammatory cells, resulting in endothelial dysfunction, is presented in a histological study of vascular tissue of postmortem human kidney, bowel, and lung in COVID-19 patients^[4]^ (Figure 1). Moreover, the recent letter of Qanadli et al.^[5]^ describes the vascular appearance in COVID-19 patients, as “vascular thickening”, an observation confirmed more recently by Bai et al.^[6]^. Together, these findings suggest that there is an effect of COVID-19 on vascular function and, therefore, that this virus plays an important role in vascular dysfunction and inflammation in COVID-19 patients. In fact, due to the complex cardiovascular features of COVID-19 patients, major cardiology organizations, including the European Society of Cardiology and American College of Cardiology, have issued bulletins for the diagnosis, management, and clinical guidance of cardiovascular disease during the COVID-19 pandemic^[7,8]^.

Angiotensin-converting enzyme 2 (ACE2), which presents high homology with angiotensin converting enzyme (ACE), is a key enzyme in the renin-angiotensin-aldosterone system (RAAS). Moreover, ACE2 is also a critical receptor for SARS-CoV-2 infections^[9]^. These findings indicate that ACE2 might be crucial for the human infection of SARS-CoV-2 and for the progression and prognosis of COVID-19. Organs expressing a high level of ACE2 are potential targets of SARS-CoV-2 infection. The subsequent tissue damage and inflammation cause severe complications that may worsen the clinical outcome^[9,10]^. Interestingly, Donoghue et al.^[11]^ demonstrated that ACE2 is expressed in the endothelium of most human intramyocardial vessels including capillaries, venules, medium-sized coronary arteries, and arterioles. In this study, the authors showed immunostaining of vascular smooth muscle cells and focal staining in the adventitia of some larger vessels. Furthermore, ACE2 expression of endothelial cells lining the lumen of the human saphenous vein (SV), commonly used as a coronary artery bypass graft, has been demonstrated as well as of those endothelial cells of the adventitial vasa vasorum^[12]^. However, while ACE2 immunoreactivity of the luminal endothelium was not observed in a separate study, it was clearly present in the medial layer of the internal mammary artery (IMA), the gold standard coronary artery bypass conduit^[13]^. However, both studies were performed on small sample numbers and on patients receiving drug therapies that might influence the results.

**Fig. 2:**
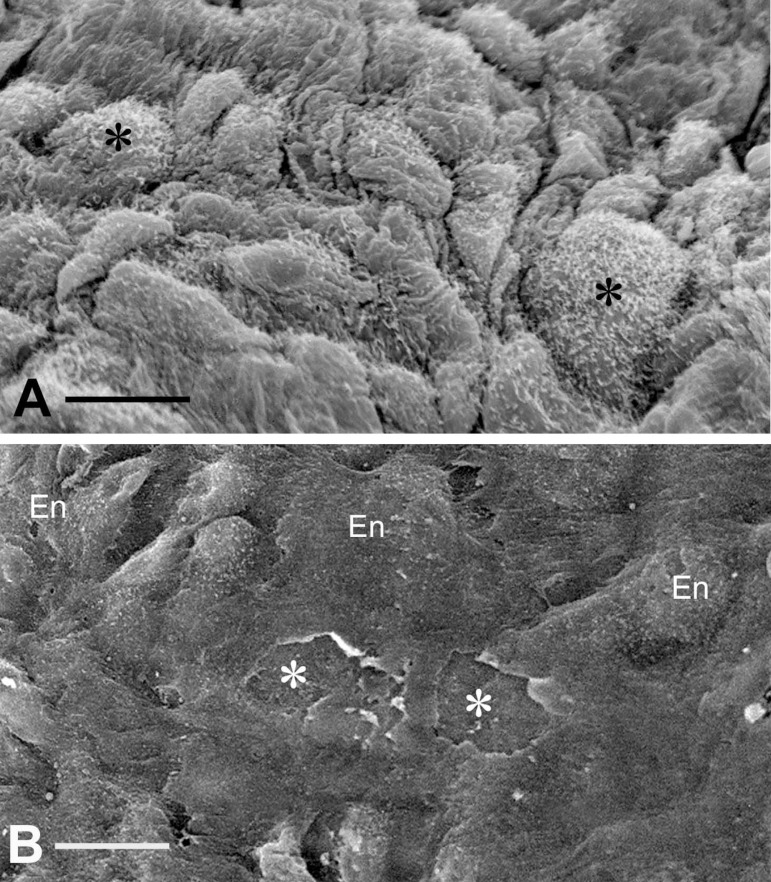
Endothelium (En) of no-touch technique (A) and conventional technique (B) saphenous vein grafts for coronary artery bypass grafting. In A, note healthy intact En with the apical microvilli (asterisks). Bar: 10 µm. In B, note deformed En, endothelial lesions, and exposure of subendothelial connective tissue/matrix (asterisks). Bar: 20 µm. (From Vasilakis et al.^[20]^, which is acknowledged).

Coronary artery bypass grafting (CABG) is performed on over one million patients a year in order to restore myocardial blood supply. CABG involves mainly the use of SV and IMA as graft material in the stenosed coronary arteries. SV is the most widely used coronary bypass conduit in addition to IMA in more than 90% of CABG worldwide^[14]^. However, SV graft patency is inferior to IMA graft patency, with approximately 50% of SV grafts failing within 10 years and patients requiring regrafting to restore myocardial blood supply^[15]^. As an *in situ* graft, IMA is relatively undamaged on exposure whereas, when harvested conventionally, SV is subjected to considerable vascular trauma. The conventional method of preparing SV for CABG in most cardiac centers is that originally described by Favaloro^[16]^. When harvesting the SV in this way, considerable vascular damage is inflicted, particularly to the adventitia, and this affects graft quality and performance. In addition, a high proportion of SVs go into spasm at harvesting due to surgical trauma and direct handling of the vein by surgical instruments and this is overcome using intraluminal saline distension at pressures approaching 700 mmHg^[17]^. These conditions cause damage to the endothelium (Figure 2) of both the SV lumen and vasa vasorum^[18-24]^. This vascular damage may not only have an immediate effect on graft patency, for example, the vasospasm that occurs at harvesting and graft implantation, but may also stimulate processes influencing thrombotic occlusion, neointimal hyperplasia, and accelerated atherosclerosis, thus affecting both medium- and long-term graft performance.

Conventional SV grafts exhibit varying degrees of vascular damage that impact on graft patency whereas those harvested by the atraumatic, no-touch (NT) technique provide superior grafts that retain their normal architecture^[25,26]^. Access to samples of both conventional and NT SV graft material offers the ability to compare damaged (conventional) with normal, undamaged, SV (NT) used in patients undergoing CABG. Of particular relevance to the link between the endothelium and COVID-19 are the ultrastructural changes observed in conventional compared to samples of normal/NT SV. At the light microscope level, pronounced regions of endothelial denudation were identified in sections of conventional SV compared with normal/NT SV where the endothelium remains virtually intact^[27]^. Such endothelial damage exposes the intimal basement membrane (Figure 2B), providing potential sites for increased platelet aggregation, increased incidence of thrombotic occlusion, and graft failure^[25]^. Endothelial damage and/or dysfunction modifies the release and equilibrium between endothelium-derived substances such as nitric oxide (NO), prostanoids, and endothelin-1 (ET-1). In many situations, endothelial dysfunction leads to increased levels of ET-1^[28]^ and thromboxane^[29]^, while there is a significant decrease in levels of NO and prostacyclin (PGI_2_)^[30-32]^. Therefore, preventing or reducing endothelial damage in SV at harvesting would be expected to have a beneficial impact on graft patency. In addition, various *in vitro* experimental studies show that perivascular adipose tissue surrounding SV provides an additional source of PGI_2_ and NO^[33,34]^, potentially preventing platelet aggregation, thrombus formation, and vasospasm and thus confirming the beneficial effect of retaining SV perivascular adipose tissue on endothelial function when harvesting the SV for CABG^[25]^.

**Fig. 3:**
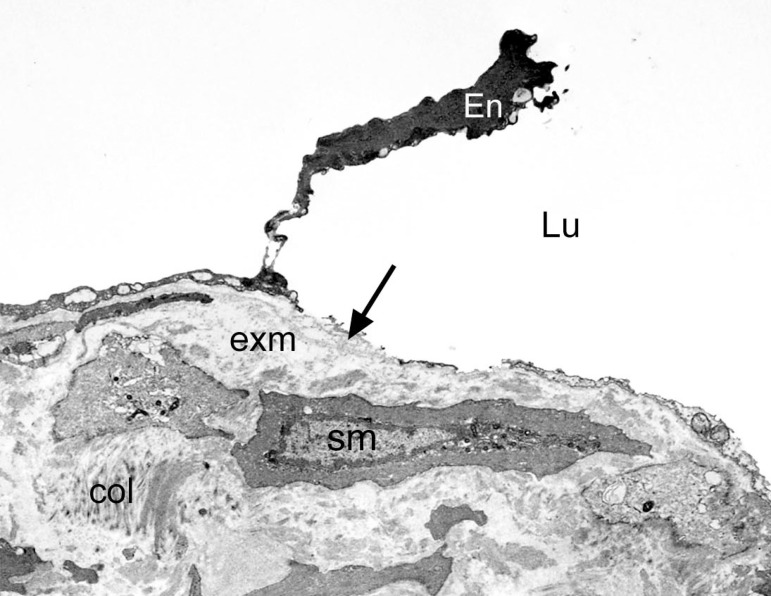
A thinned cell of endothelium (En) containing mostly nucleus or ‘dense’ cytoplasm is protruding into the saphenous vein lumen (Lu). Arrow points to exposed subendothelial extracellular matrix (exm). sm=vascular smooth muscle; col=collagen. X 5,000. (From Ahmed et al.^[19]^, which is acknowledged).

At the ultrastructural level, Ahmed et al.^[19]^ examined segments of SV harvested for CABG using conventional and NT techniques. The results showed better preservation of the ultrastructure of normal/NT SV grafts compared to those harvested conventionally. This included better preservation of the lumenal endothelium, endothelial cells of the vasa vasorum, and medial smooth muscle cells. The nuclei of endothelial cells of normal/NT SV protrude towards the lumen with junctions between the cells. Smooth muscle cells, elastin, and collagen fibers were also observed. At higher magnification, the nucleus, endoplasmic reticulum, Golgi apparatus, mitochondria, and Weibel-Palade bodies were evident^[19]^. There were dramatic morphological changes to a high proportion of endothelial cells in conventionally harvested SV (Figure 3). Polymorphism of the endothelium, cells with “dark” cytoplasm, or very thin cell processes protruding to the vein lumen were present. Some endothelial cells appeared “squashed” from the lumenal side with others “thinned” and containing mostly nucleus or “dense” cytoplasm protruding into the SV lumen. Also, various squamous endothelial cell fragments were abundant in electron-transparent cytoplasmic vesicles and extracellular matrix.

Certain interesting features were also observed when comparing normal/NT and conventional SV vasa vasorum in the tunica media. Vasa vasorum of normal/NT SV had typical-appearing endothelial cells surrounding an open lumen often containing characteristically shaped erythrocytes. By contrast, in conventional SV, clumps of erythrocytes were seen obstructing the lumen of the vasa vasorum. Interestingly, a similar situation has recently been observed in postmortem samples from patients with COVID-19 in China where “…prominent erythrocyte aggregates obstructing the lumen of capillaries…contribute to acute kidney injury, systemic hypoxia and abnormal coagulation…”^[35]^.

Since it was reported that ACE2 is an active site of SARS-CoV-2 infections and is expressed in vascular endothelium, various comments have been raised regarding the involvement of endothelial dysfunction in the pathophysiology of cardiovascular complications induced by COVID-19 infection^[4,10]^. It has been suggested that increased risk of mortality in COVID-19 infected patients with diabetes, hypertension, and obesity might be related with the progression of endothelial dysfunction in these patients^[10]^. Early findings of Varga et al.^[4]^, on endothelial cell infection and endotheliitis in patients with COVID-19, suggest that vascular inflammation induces endothelial dysfunction that also causes increased vascular permeability and thrombin generation. Therefore, dysfunctional endothelium may become procoagulant. It has been demonstrated that increased levels of pro-inflammatory cytokines, including interleukin 1β, interleukin 6 (IL-6), and tumor necrosis factor alpha (TNFα), in patients with COVID-19 can induce upregulation of procoagulants^[36]^. Overall, SARS-CoV-2 binds ACE2 receptor on endothelial cells causing inflammatory cell infiltration, endothelial cell apoptosis, and vascular prothrombotic effects^[4,10]^.

**Fig. 4:**
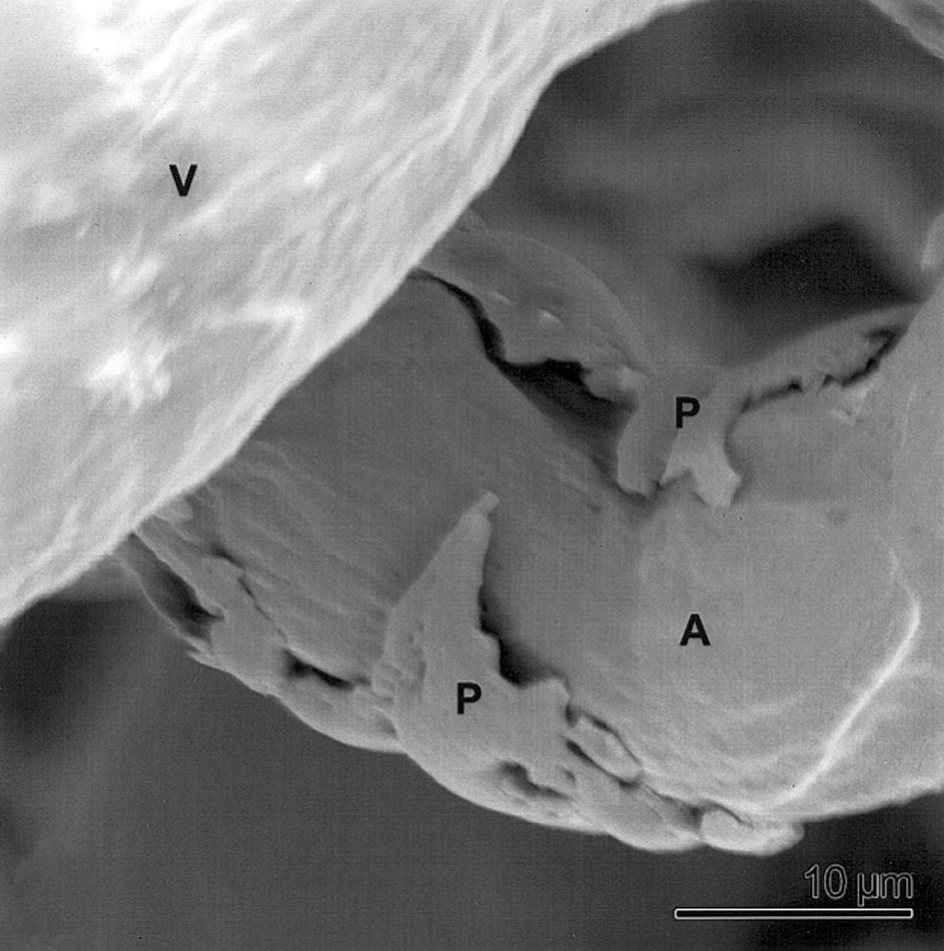
Human saphenous vein vasa vasorum. An example of plasticized (corrosion cast) myocyte/pericyte (P) embracing and constricting an artery (A). V=vein. Bar=10 µm. (From Lametschwandtner et al.^[40]^, which is acknowledged).

To date, there is no information regarding how COVID-19 affects coronary artery bypass graft vessels, in particular SV graft patency. In CABG patients, SARS-CoV-2 could bind ACE2 in SV and, therefore, influence the incidence of graft failure through an increase in vascular inflammation and vascular and endothelial dysfunction. Moreover, the inflammatory effects of cytokines also result in activated vascular endothelial cells and endothelial injury with resultant prothrombotic properties^[37]^. Also, the increased release of pro-inflammatory cytokines, including TNFα and IL-6, from human SV under inflammatory conditions has been shown, suggesting the possible interaction of vascular inflammation, endothelial dysfunction, and the risk of thrombosis in SV graft failure^[38]^. Additionally, Chen et al.^[39]^ demonstrated that SARS-CoV-2 in the human heart attacks pericytes, causing capillary endothelial dysfunction, therefore favoring a microcirculation disorder. The authors suggest that these patients are more vulnerable to cardiac damage by COVID-19. Interestingly, the presence of pericytes on arteries and capillaries of human SV vasa vasorum system has been demonstrated using plasticized corrosion casts^[40]^ (Figure 4). Based on these findings, we suggest that SARS-CoV-2 can invade pericytes of the adventitial vasa vasorum of SV leading to vascular dysfunction - *e.g*. by altering pericyte contractile properties^[41]^, causing a reduction in diameter and blood flow of vasa microvessels, resulting in medial ischemia. Damage to vasa vasorum in vein grafts results in vessel wall hypoxia with subsequent neointima formation, similar to the observation described in arteries where occlusion of vasa vasorum leads to neointima formation and atherosclerosis^[42-44]^. In addition, the physiological properties of pericytes, including those of the vasa vasorum of human SV, are now recognized as cells involved in regenerative processes with a potential application for the treatment of CAD or other forms of heart damage^[45-47]^. Therefore, it seems desirable that SV used for myocardial revascularization in patients undergoing CABG should not be stripped of the adventitial vasa vasorum system and adjacent pericytes. Furthermore, Chen et al.^[39]^ described a pericyte/endothelium/COVID-19/ACE2 connection in cardiac injury, suggesting that “the endothelial cell of capillary and part of venules may play an essential role in myocardial microcirculation”. Thus, it might be suggested that a similar axis exists for the endothelium of both the lumen and vasa vasorum of the SV. On this basis, as in the human heart, “SARS-CoV-2 infection might attack pericytes and cause capillary/vasa vasorum endothelial cell dysfunction, thus inducing micro-circulation disorder”. When using conventional SV harvesting are surgeons “using a damaged graft to repair a damaged heart” and what effect does damaging the endothelium have on CABG patients infected with COVID-19?

Overall, it is now suggested that vascular inflammation, endothelial dysfunction, and thrombosis might be some of the important key factors in the pathophysiology of COVID-19-induced cardiovascular effects. Therefore, immunomodulators like TNFα inhibitors in addition to anticoagulants and antiplatelet agents are recommended in the treatment of COVID-19^[10]^. Furthermore, a recent study demonstrated that RAAS inhibitors (ACE inhibitors and angiotensin-receptor blockers) do not have negative impact on patients with COVID-19. There are also recent observations and discussions regarding the imbalance in RAAS caused by COVID-19 that might be protected by the use of ACE inhibitors in COVID-19 patients^[48]^. However, these findings and suggestions should be investigated in further clinical trials.

Varga et al.^[4]^ suggest that ACE inhibitors and statins might have beneficial effects in preventing endothelial dysfunction in patients with COVID-19. This is a plausible hypothesis which suggests that therapeutic strategies focusing on endothelium-derived vasoactive factors may provide a timely opportunity for preventing or diminishing the development of endothelial dysfunction induced by COVID-19. In addition to ACE inhibitors and statins, ET-1 antagonists^[49]^ and NO donors^[50,51]^ might also be considered as compounds with the potential to improve SV graft performance in CABG patients infected by COVID-19. Antithrombotic strategies, including anticoagulants and antiplatelet agents in addition to RAAS inhibitors and immunomodulators, might also have beneficial effects on COVID-19-induced cardiovascular complications in CABG patients.

## CONCLUSION

In this special article, we provide an overview regarding the possible effect of COVID-19 on patency rate of coronary artery bypass grafts, mainly with reference to the SV. We propose a therapeutic potential of focusing on endothelial dysfunction, vascular inflammation, and thrombosis, not just of ACE inhibitors but of other repurposed compounds that alone, or in combination with other drugs, might be used for protecting bypass grafts in COVID-19 infected CABG patients. Therefore, these drug therapies should be considered in future clinical and experimental studies focusing on COVID-19/endothelial and vascular functions in relation to patency rates of coronary artery bypass grafts.

**Table:** 

Authors' roles & responsibilities	
GT	Substantial contributions to the conception or design of the work; drafting the work or revising it critically for important intellectual content; agreement to be accountable for all aspects of the work in ensuring that questions related to the accuracy or integrity of any part of the work are appropriately investigated and resolved; ﬁnal approval of the version to be published.
AL	Substantial contributions to the conception or design of the work; drafting the work or revising it critically for important intellectual content; agreement to be accountable for all aspects of the work in ensuring that questions related to the accuracy or integrity of any part of the work are appropriately investigated and resolved; ﬁnal approval of the version to be published.
MRD	Substantial contributions to the conception or design of the work; drafting the work or revising it critically for important intellectual content; agreement to be accountable for all aspects of the work in ensuring that questions related to the accuracy or integrity of any part of the work are appropriately investigated and resolved; ﬁnal approval of the version to be published.
